# Characterisation of malignant peripheral nerve sheath tumours in neurofibromatosis-1 using heterogeneity analysis of ^18^F-FDG PET

**DOI:** 10.1007/s00259-017-3733-1

**Published:** 2017-06-07

**Authors:** Gary J. R. Cook, Eitan Lovat, Muhammad Siddique, Vicky Goh, Rosalie Ferner, Victoria S. Warbey

**Affiliations:** 1 Cancer Imaging Department, Division of Imaging Sciences and Biomedical Engineering, King’s College London, London, SE1 7EH UK; 2 Guy’s, Kings and St Thomas’ Medical School, King’s College London, London, UK; 3 National Neurofibromatosis Service, Department of Neurology, Guys & St Thomas’ NHS Foundation Trust, London, UK

**Keywords:** ^18^F-FDG pet, Heterogeneity, Standardised uptake value, Neurofibromatosis-1, Malignant peripheral nerve sheath tumour

## Abstract

**Purpose:**

Measurement of heterogeneity in ^18^F-fluorodeoxyglucose (^18^F-FDG) positron emission tomography (PET) images is reported to improve tumour phenotyping and response assessment in a number of cancers. We aimed to determine whether measurements of ^18^F-FDG heterogeneity could improve differentiation of benign symptomatic neurofibromas from malignant peripheral nerve sheath tumours (MPNSTs).

**Methods:**

^18^F-FDG PET data from a cohort of 54 patients (24 female, 30 male, mean age 35.1 years) with neurofibromatosis-1 (NF1), and clinically suspected malignant transformation of neurofibromas into MPNSTs, were included. Scans were performed to a standard clinical protocol at 1.5 and 4 h post-injection. Six first-order [including three standardised uptake value (SUV) parameters], four second-order (derived from grey-level co-occurrence matrices) and four high-order (derived from neighbourhood grey-tone difference matrices) statistical features were calculated from tumour volumes of interest. Each patient had histological verification or at least 5 years clinical follow-up as the reference standard with regards to the characterisation of tumours as benign (*n* = 30) or malignant (*n* = 24).

**Results:**

There was a significant difference between benign and malignant tumours for all six first-order parameters (at 1.5 and 4 h; *p* < 0.0001), for second-order entropy (only at 4 h) and for all high-order features (at 1.5 h and 4 h, except contrast at 4 h; *p* < 0.0001–0.047). Similarly, the area under the receiver operating characteristic curves was high (0.669–0.997, *p* < 0.05) for the same features as well as 1.5-h second-order entropy. No first-, second- or high-order feature performed better than maximum SUV (SUVmax) at differentiating benign from malignant tumours.

**Conclusions:**

^18^F-FDG uptake in MPNSTs is higher than benign symptomatic neurofibromas, as defined by SUV parameters, and more heterogeneous, as defined by first- and high-order heterogeneity parameters. However, heterogeneity analysis does not improve on SUVmax discriminative performance.

## Introduction

Neurofibromatosis-1 (NF1) is an inherited disease characterised by multiple neurofibromas in which there is an increased risk of malignant transformation to malignant peripheral nerve sheath tumours (MPNSTs) [1]. Non-invasive differentiation of benign symptomatic neurofibromas from those with malignant transformation is a clinical challenge. Standardised uptake value (SUV) or tumour-to-liver ratio measurements from ^18^F-fluorodeoxyglucose (^18^F-FDG) positron emission tomography (PET) have previously been described as an accurate method to detect MPNSTs in this patient group [2–6]. Qualitative scoring of heterogeneity of ^18^F-FDG PET on a three-point scale has also been described where MPNSTS displayed a more heterogeneous uptake of tracer with similar discriminatory power to maximum SUV (SUVmax) [7].

There is increasing interest in the quantitative measurement of heterogeneity in medical images of cancer patients, including computed tomography (CT), magnetic resonance imaging (MRI) and PET. There is evidence that the use of heterogeneity parameters may improve characterisation, segmentation, prognostication and therapy response assessment compared to standard metrics such as size or lesion activity [8–12]. The most commonly used methods involve the measurement of statistically based parameters including first-, second- and high-order features. First-order features include global parameters such as SUV but also heterogeneity parameters, such as standard deviation (SD), first-order entropy and first-order uniformity. These are derived from intensity volume histograms of a tumour volume of interest (VOI) [8, 10, 12]. Second-order features, most often derived from grey-level co-occurrence matrices (GLCM), measure the relationship between pairs of voxels [13] and high-order features, most often derived from neighbourhood grey-tone difference matrices (NGTDM), measure the relationship between three of more voxels in the same or adjacent planes [14].

Our hypothesis was that quantitative heterogeneity parameters from ^18^F-FDG PET could improve differentiation of benign symptomatic neurofibromas from MPNSTs compared to standard PET metrics such as SUV and our aim was to compare discriminative ability in a retrospective cohort of patients with NF1 whose tumours had been well-characterised.

## Patients and methods

A cohort of 54 consecutive patients with NF1 and clinical suspicion of malignant transformation of symptomatic neurofibromas, referred from our national neurofibromatosis service for ^18^F-FDG PET/CT scans, was identified. There were 30 male (mean age 34.7 years, range 12 to 73 years) and 24 female patients (mean age 35.5 years, range 9 to 86 years). An institutional review board waiver was obtained for retrospective analysis of these data.

^18^F-FDG PET/CT scans were all acquired to the same protocol in the same institution on one of two scanners (Discovery VCT or DST, GE Healthcare, Chicago, IL, USA) which were cross-calibrated to within 3% [15]. Patients were fasted for at least 6 h prior to administration of 350 (+/− 10%) MBq ^18^F-FDG (scaled to body weight/70 in paediatric patients) and were only acquired if the blood glucose measurement was less than 10 mmol/l. Scans were acquired according to the institutional standard clinical protocol for NF1 patients with an acquisition at approximately 1.5 h (101.5 +/− 15 min) from the upper thigh to the base of skull followed by an acquisition at approximately 4 h (251.7 +/− 18.4 min) of the symptomatic tumour site only, all at 5 min per bed position [2]. Images were all reconstructed using an ordered subset expectation maximisation algorithm (2 iterations, 20 subsets) with a reconstructed slice thickness of 3.27 mm and pixel size 4.7 mm. The CT component of the scans was acquired at 120 kVp and 65 mAs without administration of oral or intravenous contrast agent.

**Fig. 1:**
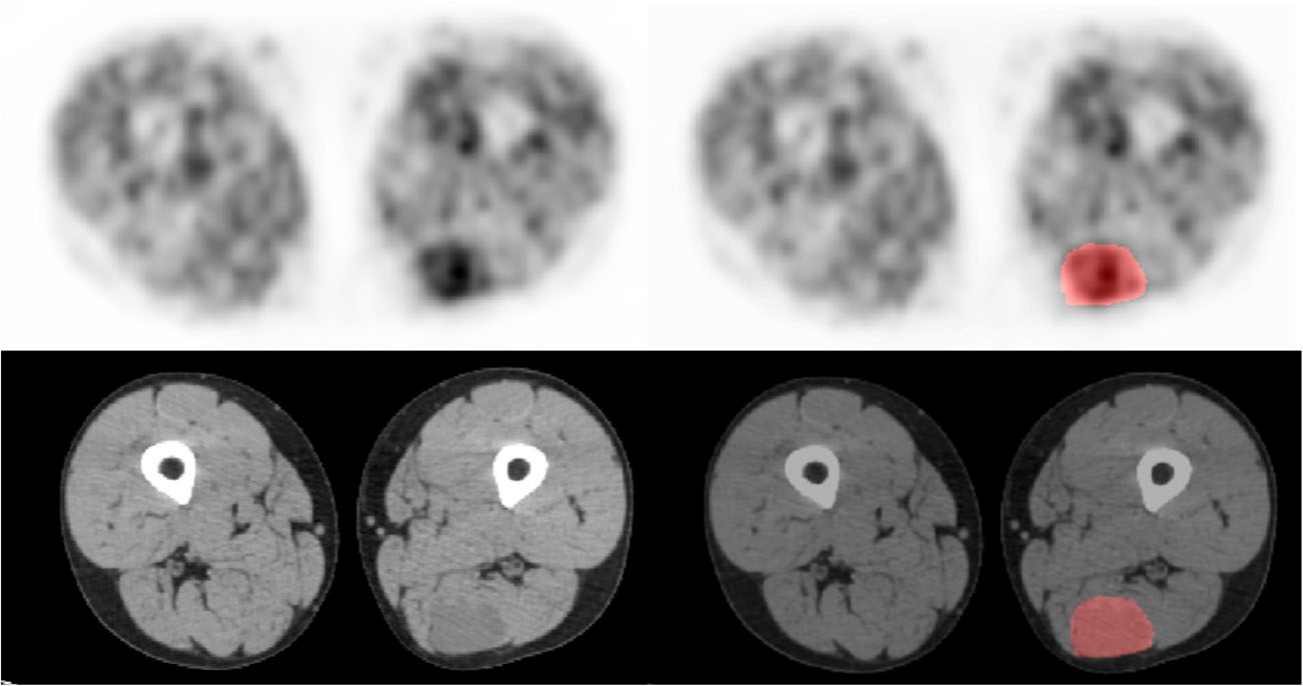
^18^F-FDG PET and CT (*left*) with corresponding images with ROIs (*right*). A symptomatic but benign left posterior thigh neurofibroma (SUVmax = 2.83)

The reconstructed PET datasets were imported into in-house texture analysis software implemented in MATLAB (Release 2016a, The MathWorks, Inc., Natick, MA, USA). Voxel intensities within the symptomatic tumour VOI were resampled to yield 64 discrete bins. Whilst most patients had multiple neurofibromas, only the symptomatic tumours were analysed. Since many of the tumours showed only low-grade FDG uptake, it was not possible to adequately segment the tumour regions directly from the PET data by freehand or by using semi-automated methods such as percentage threshold or fuzzy locally adaptive Bayesian methods [16]. Regions of interest were, therefore, drawn on the corresponding CT images where tumours were more easily defined (Fig. 1) by an experienced operator with radiology and nuclear medicine training and over 20 years experience. To assess inter-observer variability, a random subset of 16 patients had VOIs defined on 1.5- and 4-h scans by a separate operator blinded to the initial observer measurements and clinical data.

As well as SUVs (mean, maximum and peak, all normalised to body weight in kilogrammes), three first-order (SD, entropy and uniformity), four second-order GLCM parameters (contrast, entropy, uniformity and homogeneity) and four high-order NGTDM parameters (coarseness, contrast, busyness and complexity) were calculated from the resulting VOIs. Second-order features were calculated from GLCMs measuring the grey-level distribution between pairs of voxels and high-order features were derived from three-dimensional matrices taking into consideration neighbouring voxels in adjacent planes. All these features have been previously described in detail [13, 14] and the chosen parameters have previously shown utility and/or robustness when used in clinical ^18^F-FDG PET data of cancers [17–22].

Statistical analysis was performed using SPSS (v22, Chicago, IL, USA) and MedCalc (v16.8.4, Ostend, Belgium) software. The data distributions were tested for normality using the Shapiro–Wilk test. As data were not normally distributed differences between benign and malignant tumours were tested with the Mann–Whitney U test for each parameter and correlations between parameters with Spearman correlation. Receiver operator characteristic (ROC) curves were also used to compare the ability of each parameter to classify tumours as benign or malignant and the area under ROC curves (AUROC) were calculated. Comparisons between AUROC were made as described by DeLong et al. [23]. Separate assessment was made by combining SUVmax with other parameters that did not show a correlation with SUVmax. Statistical significance was assumed when *p* < 0.05. Inter-observer variation was assessed with intra-class correlation coefficients (ICCs).

## Results

Thirty patients had benign tumours and 24 had MPNSTs confirmed either histologically (*n* = 30) or by at least 5 years of follow-up (*n* = 24). Thirty-six symptomatic tumours were on the trunk and 18 in the extremities.

Good inter-observer agreement was found for measurement of all parameters with ICC varying from 0.86 (NGTDM contrast and GLCM contrast) to 1.0 (SUVmax and SUVpeak) on 1.5-h and 4-h scans. Median (and range) malignant and benign tumour volumes were 60.0 cm^3^ and 23.2 cm^3^, respectively (8.3–303.9 and 3.3–164.1 cm^3^, respectively, *p* = 0.004).

**Fig. 2:**
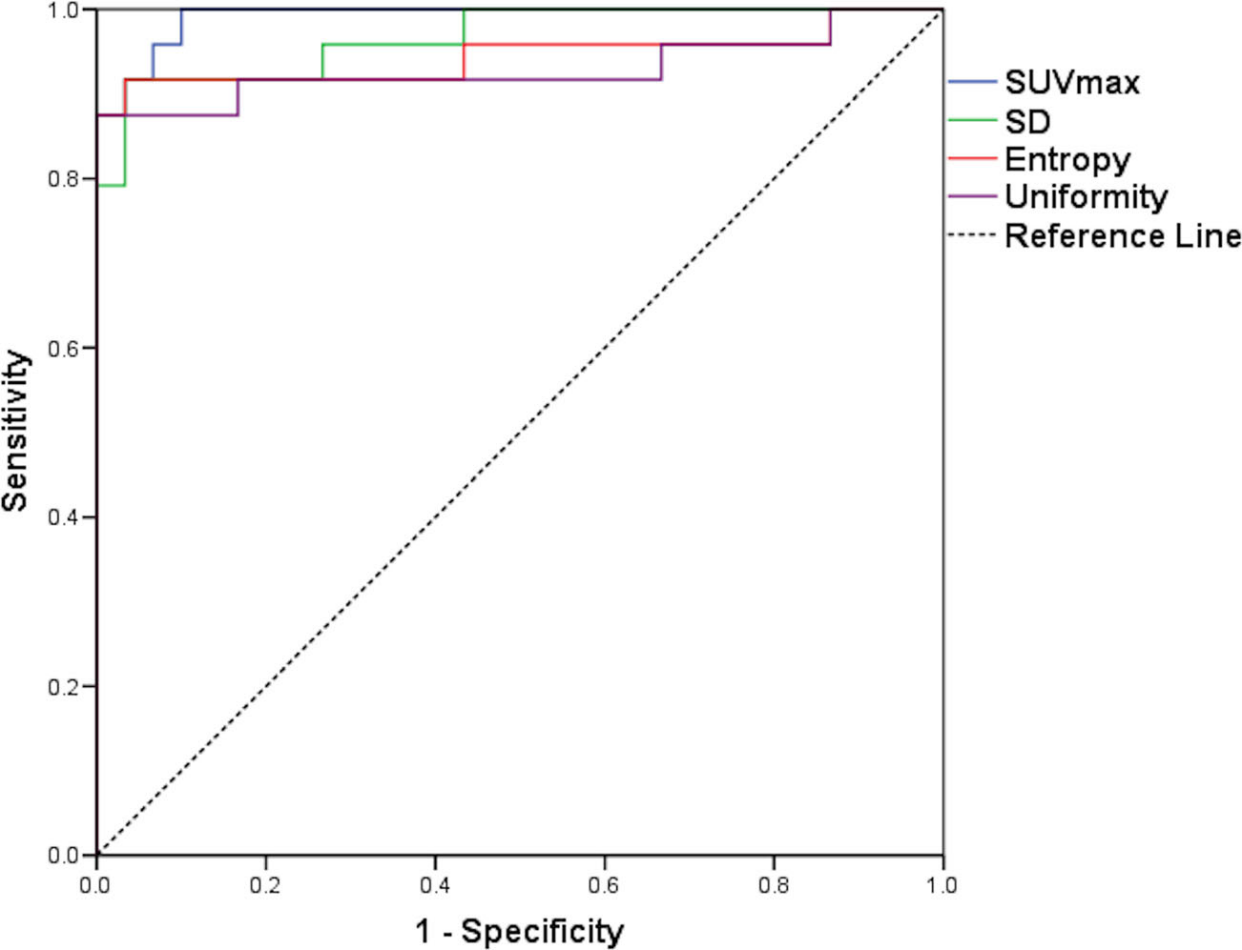
ROC curves for SUVmax and first-order parameters (SD, entropy and uniformity) at 1.5 h. See Table 2 for AUROCs. There was no statistically significant difference between SUVmax AUROC and the other first-order parameter AUROCs (all *p* > 0.05)

**Fig. 3:**
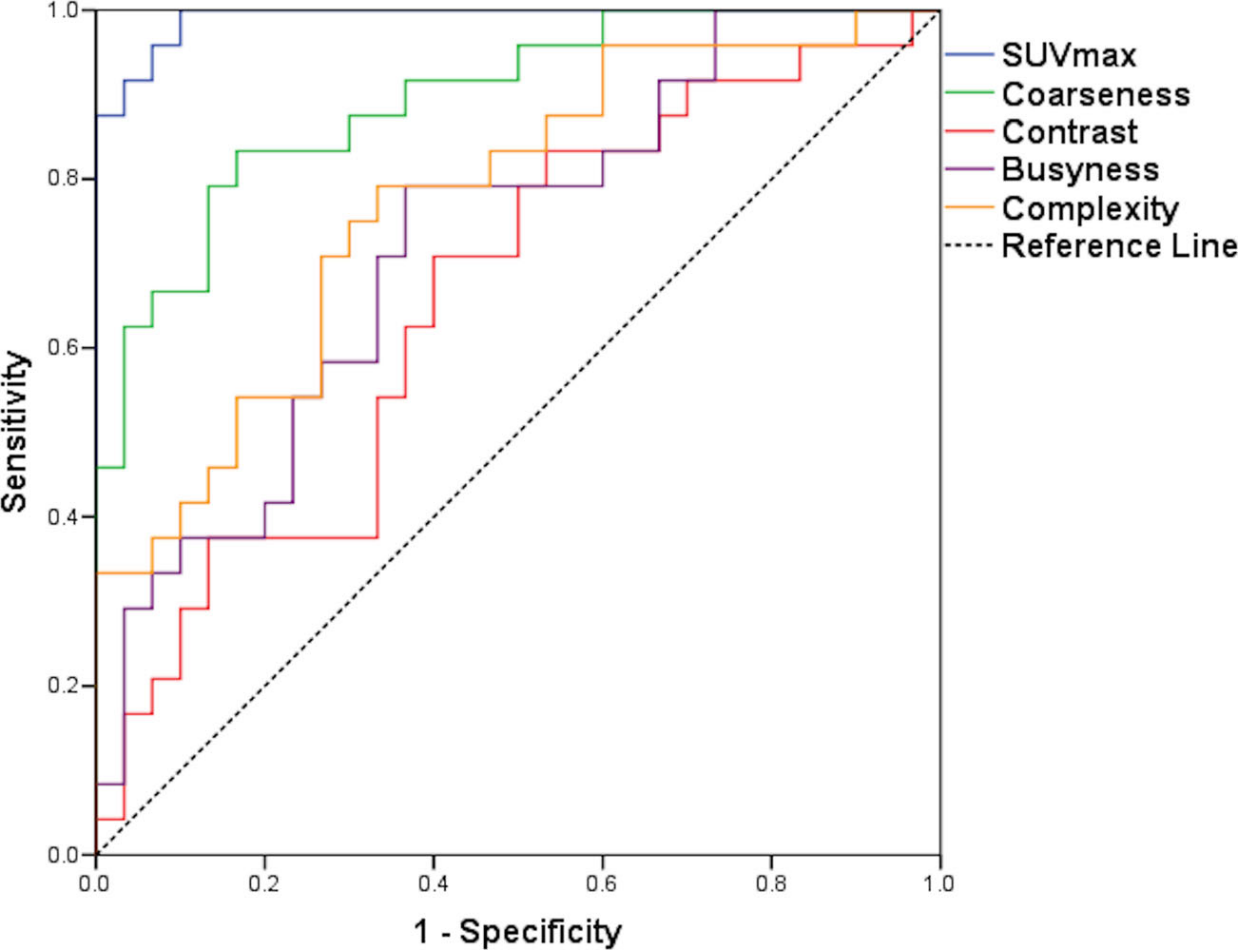
ROC curves for SUVmax and high-order parameters (coarseness, contrast, complexity, busyness) at 1.5 h. See Table 2 for AUROCs. There was a statistically significant difference between SUVmax AUROC and the other high-order parameter AUROCs (coarseness *p* = 0.019, contrast *p* < 0.0001, busyness *p* = 0.0009, complexity *p* = 0.0002)

On 1.5-h scans, there was a significant difference between benign and malignant tumours for all SUV and other first-order parameters, for none of the second-order parameters and for all four high-order parameters. At 4 h, the results were the same, except second-order entropy was significantly different; high-order contrast was not (Table 1). Only percentage change SUVmean and SUVpeak showed significant differences between benign and malignant lesions (Table 1). For ROC analysis, SUV and other first-order parameters, second-order entropy and all high-order parameters showed ability to discriminate at 1.5 and 4 h (except high-order contrast at 4 h; Table 2). SUVmax showed the highest AUROC at 1.5 h (0.992) and SUVpeak at 4 h (0.997), closely followed by SUVmax (0.996). SD showed the best discrimination from the other first-order features (0.967 and 0.99 at 1.5 and 4 h, respectively; Fig. 2). Coarseness showed the best discrimination from the high-order features (0.894 and 0.888 at 1.5 and 4 h, respectively; Table 2; Fig. 3). The percentage change in SUVmean and SUVpeak showed some discriminatory ability (AUROC 0.722 and 0.688, respectively; Table 2).

**Table 1:** Differences between benign and malignant tumours for each heterogeneity parameter at 1.5 and 4 h post-injection of ^18^F-FDG and for percentage change in values between 1.5 and 4 h

Parameter	Benign (1.5 h) median (range)	Malignant (1.5 h) median (range)	*p* value benign vs malignant (1.5 h)	Benign (4 h) median (range)	Malignant (4 h) median (range)	*p* value benign vs malignant (4 h)	% change median (range)	*p* value benign vs malignant (% change)
SUVmax	2.28 (0.89–4.49)	7.01 (3.12–30.8)	<0.0001	1.98 (0.8–4.66)	8.24 (3.56–30.8)	<0.0001	−6.3% (−133.3–56.8)	0.082
SUVmean	1.06 (0.5–1.85)	3.07 (1.34–8.89)	<0.0001	0.81 (0.41–1.44)	3.1 (1.24–9.1)	<0.0001	12.3% (−168.8–52.4)	0.005
SUVpeak	1.66 (0.72–3.04)	5.98 (2.41–23.38)	<0.0001	1.43 (0.7–2.65)	6.14 (2.58–23.3)	<0.0001	4.1% (−118.6–53.4)	0.019
SD	0.28 (0.12–0.82)	1.26 (0.35–5.05)	<0.0001	0.29 (0.12–0.72)	1.33 (0.43–5.12)	<0.0001	−8.5% (153.5–55.0)	0.17
First-order entropy	0.68 (0.17–1.18)	1.56 (0.44–4.76)	<0.0001	0.69 (0.12–1.15)	1.62 (0.67–2.99)	<0.0001	−4.6% (−186.0–71.1)	0.6
First-order uniformity	0.56 (0.39–0.92)	0.25 (0.04–0.78)	<0.0001	0.54 (0.37–0.95)	0.245 (0.06–0.65)	<0.0001	5.3% (−638.6–58.3)	0.37
GLCM contrast	36.6 (15.9–107.5)	36.1 (8.4–140.1)	0.78	42.2 (11.7–146.2)	39.3 (5.4–98.5)	0.35	--6.4% (−207.5–70.6)	0.42
GLCM entropy	5.79 (4.39–6.22)	6.05 (4.7–6.46)	0.12	5.67 (4.91–6.29)	5.88 (4.17–6.44)	0.034	1.3% (−35.1–15.6)	0.81
GLCM uniformity	0.02 (0.01–0.05)	0.02 (0.01–0.06)	0.99	0.02 (0.01–0.05)	0.02 (0.01–0.1)	0.54	−4.8% (−177.7–75.8)	0.55
GLCM homogeneity	0.72 (0.0.53–0.81)	0.73 (0.55–0.89)	0.32	0.73 (0.56–0.85)	0.74 (0.5–0.91)	0.44	−0.7% (−19.1–21.0)	0.19
NGTDM coarseness	5.85 (4.26–10.59)	10.28 (5.47–17.37)	<0.0001	6.15 (3.98–10.71)	9.16 (6.06–15.3)	<0.0001	3.6% (−66.1–42.2)	0.53
NGTDM contrast	0.17 (0.05–0.59)	0.14 (0.02–0.43)	0.047	0.15 (0.06–0.46)	0.107 (0.008–0.343)	0.23	10.6% (−212.8–68.9)	0.9
NGTDM busyness	3.2 (0.67–22.47)	6.82 (1.68–32.89)	0.005	3.66 (0.78–19.21)	8.54 (1.44–33.32)	0.013	−10.3% (−314.8–89.2)	0.58
NGTDM complexity	0.19 (0.02–2.0)	0.04 (0.01–0.63)	0.001	0.17 (0.03–1.55)	0.043 (0.005–0.598)	<0.0001	−5.3% (−1354–88.6)	0.97
SUVmax/NGTDM contrast	12.6 (2.1–55.8)	56.4 (20.2–1137.2)	<0.001	14.8 (2.9–77.3)	77.3 (26.0–3685.9)	<0.001	−12.9% (−294.7–82.8)	0.54

*SUV* standardised uptake value, *SD* standard deviation, *GLCM* grey-level co-occurrence matrix, *NGTDM* neighbourhood grey-tone difference matrix

**Table 2:** Area under receiver operating characteristic curves (AUROC), sensitivity, specificity, PPV, NPV and accuracy at 1.5 and 4 h post-injection of ^18^F-FDG and statistical comparison with SUVmax AUROC

Parameter	AUROC 1.5 h (CI) *p < 0.05	*p* value compared to SUVmax	Sensitivity, specificity, PPV, NPV, accuracy (1.5 h)	AUROC 4 h (CI) *p < 0.05	*p* value compared to SUVmax	Sensitivity, specificity, PPV, NPV, accuracy (4 h)	AUROC % change (CI) *p < 0.05
SUVmax	0.992 (0.977–1.0)*		1.0, 0.9, 0.89, 1.0, 0.94	0.996 (0.986–1.0)*		1.0, 0.93, 0.92, 1.0, 0.96	0.639 (0.488–0.789)
SUVmean	0.981 (0.953–1.0)*		0.92, 0.97, 0.96, 0.94, 0.94	0.989 (0.97–1.0)*		0.96, 0.93, 0.92, 0.97, 0.94	0.722 (0.577–0.868*
SUVpeak	0.987 (0.967–1.0)*		0.92, 1.0, 1.0, 0.94, 0.96	0.997 (0.99–1.0)*		0.96, 1.0, 1.0, 0.97, 0.98	0.688 (0.538–0.837)*
SD	0.967 (0.922–1.0)*	0.14	0.92, 0.97, 0.96, 0.94, 0.94	0.99 (0.973–1.0)*	0.27	0.92, 1.0, 1.0, 0.94, 0.96	0.61 (0.454–0.765)
First-order entropy	0.944 (0.867–1.0)*	0.18	0.88, 1.0, 1.0, 0.91, 0.94	0.95 (0.881–1.0)*	0.16	0.92, 1.0, 1.0, 0.94, 0.96	0.542 (0.385–0.698)
First-order uniformity	0.929 (0.842–1.0)*	0.12	0.88, 1.0, 1.0, 0.91, 0.94	0.926 (0.83–1.0)*	0.14	0.92, 0.97, 0.96, 0.94, 0.94	0.572 (0.41–734)
GLCM contrast	0.478 (0.318–0.638)	<0.0001	0.33, 0.8, 0.57, 0.6, 0.59	0.575 (0.42–0.73)	<0.0001	0.58, 0.83, 0.74, 0.71, 0.72	0.564 (0.406–0.721)
GLCM entropy	0.701 (0.55–0.853)*	0.0002	0.58, 0.87, 0.78, 0.72, 0.74	0.669 (0.519–0.819)*	<0.0001	0.58, 0.73, 0.64, 0.69, 0.67	0.519 (0.392–0.702)
GLCM uniformity	0.499 (0.337–0.66)	<0.0001	0.54, 0.3, 0.38, 0.45, 0.41	0.549 (0.392–0.706)	<0.0001	0.83, 0.33, 0.5, 0.71, 0.56	0.547 (0.392–0.702)
GLCM homogeneity	0.567 (0.407–0.726)	<0.0001	0.25, 0.97, 0.86, 0.62, 0.65	0.522 (0.364–0.681)	<0.0001	0.5, 0.67, 0.55, 0.63, 0.59	0.606 (0.451–0.76)
NGTDM coarseness	0.894 (0.811–0.978)*	0.019	0.83, 0.83, 0.8, 0.86, 0.83	0.888 (0.801–0.974)*	0.01	0.79, 0.87, 0.83, 0.84, 0.83	0.55 (0.393–0.707)
NGTDM contrast	0.658 (0.511–0.805)*	<0.0001	0.83, 0.47, 0.56, 0.78, 0.63	0.596 (0.442–0.75)	<0.0001	0.5, 0.67, 0.55, 0.63, 0.59	0.51 (0.353–0.666)
NGTDM busyness	0.703 (0.565–0.841)*	0.0009	0.79, 0.7, 0.68, 0.81, 0.74	0.674 (0.531–0.817)*	<0.0008	0.67, 0.67, 0.62, 0.71, 0.67	0.544 (0.383–0.706)
NGTDM complexity	0.774 (0.649–0.899)*	0.0002	0.79, 0.7, 0.68, 0.81, 0.74	0.778 (0.652–0.903)*	<0.0001	0.79, 0.70, 0.68, 0.81, 0.74	0.503 (0.343–0.662)
SUVmax/NGTDM contrast	0.96 (0.91–1.0)*	0.15	0.92, 0.93, 0.92, 0.93, 0.93	0.944 (0.89–0.999)*	0.19	0.83, 0.9, 0.87, 0.87, 0.87	0.549 (0.393–0.704)

*SUV* standardised uptake value, *SD* standard deviation, *GLCM* grey level co-occurrence matrix, *NGTDM* neighbourhood grey tone difference matrix, *PPV* positive predictive value, *NPV* negative predictive value

Most parameters showed significant correlations with SUVmax except the GLCM parameters and NGTDM contrast. GLCM parameters performed poorly in discriminating tumours and, so, were not further assessed, but the combined parameter SUVmax/NGTDM contrast was further evaluated to see if there was incremental value from this combination (Tables 1 and 2). Whilst combining the parameters in this way showed a better performance than NGTDM contrast alone, it did not show any additional value over SUVmax.

## Discussion

This study has shown that MPNSTs in patients with NF1 display greater heterogeneity of ^18^F-FDG uptake than benign symptomatic neurofibromas as measured by a number of global first-order features (including SD, entropy and uniformity) as well as local high-order features (including coarseness, contrast, busyness and complexity). To our knowledge, only qualitative measures of heterogeneity have previously been described in this scenario where a qualitative heterogeneity score showed similar sensitivity but lower specificity to SUVmax [7]. With regards to other primary soft tissue tumours, a previous study has shown that heterogeneity parameters from ^18^F-FDG PET can differentiate benign from malignant musculoskeletal tumours better than SUVmax (*p* = 0.004) [24]. Another study showed that heterogeneity of ^18^F-FDG uptake and tumour grade in sarcomas were the only independent prognostic factors predicting overall survival (*p* < 0.001 and 0.004, respectively), whereas SUVmax and tumour type were not [25]. It is hypothesised that increased heterogeneity of ^18^F-FDG uptake within tumours is related to variations in cell density and proliferation as well as more heterogeneous underlying biology including angiogenesis and hypoxia and this is why heterogeneous tumours behave more aggressively [26, 27].

Our study also showed that MPSNTs showed significantly higher ^18^F-FDG accumulation compared to benign neurofibromas as measured by SUV parameters, a finding that has been previously reported [2–4]. Whilst SUVmax showed excellent ability to discriminate MPNSTs from symptomatic benign neurofibromas as determined by AUROC (0.992, 0.996 at 1.5 and 4 h, respectively), the SUVmax AUROC was not significantly different from SD, entropy or uniformity, but was significantly higher than all high-order features (Table 2; Figs. 1 and 2). The percentage change in SUV and heterogeneity parameters between 1.5- and 4-h scans did not show any superiority in discriminating benign from malignant tumours compared to the parameters alone.

Our study is potentially limited by its retrospective nature, but our results should be representative as this was a cohort of patients referred for clinical assessment of symptomatic neurofibromas that were suspected of malignant transformation. However, it may not necessarily be possible to extrapolate the findings to other tumour types. Whilst semi-automated methods of tumour segmentation on ^18^F-FDG PET images are preferred and are likely to show even better inter-observer variation, we were unable to apply these methods due to difficulty in defining tumours with low uptake on the PET scans. Nevertheless, VOI definition from the CT images proved straightforward and with good inter-observer reproducibility. In addition, whilst all image sets were checked qualitatively for registration of the PET and CT data by an experienced observer, we cannot exclude small amounts of mis-registration due to patient movement.

## Conclusion

In patients with NF1, MPNSTs showed greater heterogeneity and greater levels of ^18^F-FDG uptake than benign symptomatic neurofibromas. First-order heterogeneity parameters were as discriminative as SUVmax. Although high-order features also showed the ability to differentiate benign and malignant tumours, these had lesser discriminatory ability compared to SUVmax.

## References

[1] Ferner RE, Gutmann DH (2002). International consensus statement on malignant peripheral nerve sheath tumors in neurofibromatosis. Cancer Res.

[2] Warbey VS, Ferner RE, Dunn JT, Calonje E, O’Doherty MJ (2009). [18F]FDG PET/CT in the diagnosis of malignant peripheral nerve sheath tumours in neurofibromatosis type-1. Eur J Nucl Med Mol Imaging.

[3] Benz MR, Czernin J, Dry SM, Tap WD, Allen-Auerbach MS, Elashoff D (2010). Quantitative F18-fluorodeoxyglucose positron emission tomography accurately characterizes peripheral nerve sheath tumors as malignant or benign. Cancer.

[4] Chirindel A, Chaudhry M, Blakeley JO, Wahl R (2015). 18F-FDG PET/CT qualitative and quantitative evaluation in neurofibromatosis type 1 patients for detection of malignant transformation: comparison of early to delayed imaging with and without liver activity normalization. J Nucl Med.

[5] Combemale P, Valeyrie-Allanore L, Giammarile F, Pinson S, Guillot B, Goulart DM (2014). Utility of 18F-FDG PET with a semi-quantitative index in the detection of sarcomatous transformation in patients with neurofibromatosis type 1. PLoS One.

[6] Salamon J, Veldhoen S, Apostolova I, Bannas P, Yamamura J, Herrmann J (2014). 18F-FDG PET/CT for detection of malignant peripheral nerve sheath tumours in neurofibromatosis type 1: tumour-to-liver ratio is superior to an SUVmax cut-off. Eur Radiol.

[7] Salamon J, Derlin T, Bannas P, Busch JD, Herrmann J, Bockhorn M (2013). Evaluation of intratumoural heterogeneity on 18F-FDG PET/CT for characterization of peripheral nerve sheath tumours in neurofibromatosis type 1. Eur J Nucl Med Mol Imaging.

[8] Chicklore S, Goh V, Siddique M, Roy A, Marsden PK, Cook GJ (2013). Quantifying tumour heterogeneity in 18F-FDG PET/CT imaging by texture analysis. Eur J Nucl Med Mol Imaging.

[9] Lambin P, Rios-Velazquez E, Leijenaar R, Carvalho S, van Stiphout RG, Granton P (2012). Radiomics: extracting more information from medical images using advanced feature analysis. Eur J Cancer.

[10] Hatt M, Tixier F, Pierce L, Kinahan PE, Le Rest CC, Visvikis D (2017). Characterization of PET/CT images using texture analysis: the past, the present… any future?. Eur J Nucl Med Mol Imaging.

[11] Alic L, Niessen WJ, Veenland JF (2014). Quantification of heterogeneity as a biomarker in tumor imaging: a systematic review. PLoS One.

[12] Bashir U, Siddique MM, Mclean E, Goh V, Cook GJ (2016). Imaging heterogeneity in lung cancer: techniques, applications, and challenges. AJR Am J Roentgenol.

[13] Haralick JM, Shanmugam K, Dinstein I (1973). Textural features for image classification. IEEE Trans Syst Man Cybern.

[14] Amadasun M, King R (1989). Textural features corresponding to textural properties. IEEE Trans Syst Man Cybern.

[15] Schleyer PJ, Baker S, Barrington SF, McWilliams S, Somer E, Marsden P (2008). Establishment of acquisition and reconstruction parameters for a GE discovery VCT PET-CT scanner. Eur J Nucl Med Mol Imaging.

[16] Hatt M, Cheze Le Rest C, Albarghach N, Pradier O, Visvikis D (2011). PET functional volume delineation: a robustness and repeatability study. Eur J Nucl Med Mol Imaging.

[17] Tixier F, Hatt M, Le Rest CC, Le Pogam A, Corcos L, Visvikis D (2012). Reproducibility of tumor uptake heterogeneity characterization through textural feature analysis in 18F-FDG PET. J Nucl Med.

[18] Hatt M, Tixier F, Cheze Le Rest C, Pradier O, Visvikis D (2013). Robustness of intratumour ^18^F-FDG PET uptake heterogeneity quantification for therapy response prediction in oesophageal carcinoma. Eur J Nucl Med Mol Imaging.

[19] Xu R, Kido S, Suga K, Hirano Y, Tachibana R, Muramatsu K (2014). Texture analysis on (18)F-FDG PET/CT images to differentiate malignant and benign bone and soft-tissue lesions. Ann Nucl Med.

[20] Cook GJ, Yip C, Siddique M, Goh V, Ahmed S, Roy A (2013). Are pretreatment 18F-FDG PET tumor textural features in non-small cell lung cancer associated with response and survival after chemoradiotherapy?. J Nucl Med.

[21] Cook GJ, O’Brien ME, Siddique M, Chicklore S, Loi HY, Sharma B (2015). Non-small cell lung cancer treated with erlotinib: heterogeneity of (18)F-FDG uptake at PET-association with treatment response and prognosis. Radiology.

[22] Forgacs A, Pall Jonsson H, Dahlbom M, Daver F, Di Franco M, Opposits G (2016). A study on the basic criteria for selecting heterogeneity parameters of F18-FDG PET images. PLoS One.

[23] DeLong ER, DeLong DM, Clarke-Pearson DL (1988). Comparing the areas under two or more correlated receiver operating characteristic curves: a nonparametric approach. Biometrics.

[24] Nakajo M, Nakajo M, Jinguji M, Fukukura Y, Nakabeppu Y, Tani A, et al. The value of intratumoral heterogeneity of (18)F-FDG uptake to differentiate between primary benign and malignant musculoskeletal tumours on PET/CT. Br J Radiol. 2015; doi:10.1259/bjr.20150552. 10.1259/bjr.20150552 PMC4743469 26337605

[25] Eary JF, O’Sullivan F, O’Sullivan J, Conrad EU (2008). Spatial heterogeneity in sarcoma 18F-FDG uptake as a predictor of patient outcome. J Nucl Med.

[26] Henriksson E, Kjellen E, Wahlberg P, Ohlsson T, Wennerberg J, Brun E (2007). 2-deoxy-2-(18F) fluoro-D-glucose uptake and correlation to intratumoural heterogeneity. Anticancer Res.

[27] Orlhac F, Thézé B, Soussan M, Boisgard R, Buvat I (2016). Multiscale texture analysis: from 18F-FDG PET images to histologic images. J Nucl Med.

